# Wearable Measurement of ECG Signals Based on Smart Clothing

**DOI:** 10.1155/2020/6329360

**Published:** 2020-01-18

**Authors:** Ming Li, Wei Xiong, Yongjian Li

**Affiliations:** ^1^Engineering Research Center of Hubei Province for Clothing Information, Wuhan Textile University, Wuhan 430200, China; ^2^Hubei Key Laboratory of Digital Textile Equipment, Wuhan Textile University, Wuhan 430200, China; ^3^Class 1112, Maple Leaf International School-Wuhan, Wuhan 410077, China

## Abstract

Smart clothing that can measure electrocardiogram (ECG) signals and monitor the health status of people meets the needs of our increasingly aging society. However, the conventional measurement of ECG signals is complicated and its electrodes can cause irritation to the skin, which makes the conventional measurement method unsuitable for applications in smart clothing. In this paper, a novel wearable measurement of ECG signals is proposed. There are only three ECG textile electrodes knitted into the fabric of smart clothing. The acquired ECG signals can be transmitted to a smartphone via Bluetooth, and they can also be sent out to a PC terminal by a smartphone via WiFi or Internet. To get more significant ECG signals, the ECG differential signal between two electrodes is calculated based on a spherical volume conductor model, and the best positions on the surface of a human body for two textile electrodes to measure ECG signals are simulated by using the body-surface potential mapping (BSPM) data. The results show that position 12 in the lower right and position 11 in the upper left of the human body are the best for the two electrodes to measure ECG signals, and the presented wearable measurement can obtain good performance when one person is under the conditions of sleeping and jogging.

## 1. Introduction

The heart, a 4-chamber organ, plays a critical role in supplying blood throughout the body. As reported by Majumder et al. in [1], heart diseases are some of the most prevalent and serious life-threatening health problems in the world and are the main cause of death for people between 44 and 64 years old; they are also the second most frequent cause of death for people between 24 and 44 years old. Therefore, the status of the heart has a great impact on our health.

The ECG signal is one of the most important parameters for monitoring the physiological status of a person, since it carries meaningful information about the person's heart. The most common method for the ECG measurement is the standard 12-lead ECG, where nine or ten gel Ag/AgCl electrodes are fixed on well-defined locations on the limbs and the torso through galvanic contact with the skin. However, there are some reasons that limit the usage of the standard 12-lead ECG measurement method in smart clothing. First, because of the complicated process, the 12-lead ECG measurement can only be performed in hospitals or clinics by professional physicians, which is not fit for those patients with chronic heart diseases who want to be cared at home and the athletes who want to monitor their health status when exercising. For the same reason, this measurement method is also not fit for soldiers, firefighters, police officers, rescue workers in mines, etc. Second, the expense and discomfort brought by the skin-contact electrodes prevent people from using the 12-lead measurement in daily life. Finally, according to the World Health Organization, more than a billion people in the world today experience disability [2], and the proportion of the world's population over 60 years will nearly double from 12% to 22% between 2015 and 2050 [3]. It is impossible to accommodate all of these people at medical centers for heart health monitoring using traditional ECG measurement. Therefore, one of the promising solutions is to design a kind of wearable measurement system based on smart clothing that can be worn on the body to record the ECG signals in the long term.

Fortunately, many efforts have recently been devoted to this field. Okada et al. in [4] developed small and light-weight wearable ECG equipment with three accelerometers. However, the ECG electrode is a conventional gel Ag/AgCl electrode. Park et al. in [5] developed an ECG monitoring system based on QUASAR's wearable ECG sensors that do not require direct contact with the skin when using. However, the sensors cannot be embedded into clothing, and therefore, they may be inconvenient to use in daily life. Arun and Alexander in [6] developed a weighted portable ECG monitoring device that used capacitive coupled electrodes embedded in an armband and could send the ECG signals obtained to a mobile phone via Bluetooth. However, the ECG electrodes may cause irritation to the skin.

Textile electrodes, made by conductive textile materials, are a new and potential choice for wearable biopotential measurement, because they can be easily integrated with clothing by some manufacturing process, such as weaving, knitting, and embroidering. And the most important thing is that the textile electrodes do not cause irritation to the human skin even when worn for a long time. Therefore, this kind of electrode is suitable for use on smart clothing to measure ECG signals. Pola and Vanhala in [7] tested and compared the performance of four different kinds of textile electrodes that were structurally different. Three of the electrodes were industrially manufactured, and the last one was handmade by embroidering conductive yarn on fabric. All the electrodes were in direct contact with the skin during measuring, and the results showed that the latter was the best in terms of accuracy. Vojtech et al. in [8] developed a kind of textile electrode that was embedded into a T-shirt with a flame retardant material and embroidered with yarn based on a mixture of polyester coated with silver nanoparticles and cotton. Caldara et al. in [9] developed a wireless low-power wearable system for use with multilead ECG monitoring where carbon-based smart textiles were used as electrodes. Yoo et al. in [10] presented a wearable ECG acquisition system implemented with a planar-fashionable circuit board- (P-FCB-) based shirt, where dry electrodes screen-printed directly on fabric enable long-term monitoring without skin irritation. However, all these devices mentioned above cannot send ECG signals obtained to remote servers and the servers also do not have the ability to store large amounts of ECG signals. Therefore, these devices have some limits for doctors to diagnose chronic heart disease for the users.

In order to realize remote diagnosis and health monitoring in the long term, some communication technologies should be used. Lee and Chung in [11] developed a kind of smart shirt that the measured physiological ECG data and physical activity data which were transmitted by an ad hoc network in IEEE 802.15.4 communication standard to a base station and a server for remote monitoring. Taji et al. in [12] presented a device that could transfer measured ECG signals to a PC, and they applied the conductive textile made by AgNy yarns as their ECG electrode. Paradiso et al. in [13] developed a wearable health care system based on knitted integrated sensors, where six fabric ECG electrodes were realized by a textile yarn, and the acquired signals were wirelessly transmitted from a Portable Patient Unit (PPU) to a remote monitoring system based on GPRS communication. However, in these systems, the acquired ECG signals cannot be transmitted to a smartphone, and therefore, the users of the system cannot see their ECG signals at any time by themselves, which may prevent them from taking necessary treatment when they feel uncomfortable with their heart.

In this paper, a novel wearable measurement of ECG signals based on smart clothing is presented. The measurement system consists of three subsystems: smart clothing, smartphone, and PC terminal. There are only three ECG textile electrodes that are knitted into the fabric of the smart clothing, and the ECG signals obtained by the electrodes can be transmitted to the smartphone via Bluetooth. Then, the ECG signals can be transmitted from the smartphone to the PC terminal via WiFi or Internet. In the PC terminal, the doctors can see large amounts of ECG signals of the users over a long period of time from the past, which will help the doctors accurately diagnose the health status of the users.

The rest of this paper is organized as follows: Section 2 describes the proposed wearable ECG measurement based on smart clothing. Then, the surface potential on the human body and the ECG differential signal between two electrodes are calculated based on a spherical volume conductor model in Section 3. The simulation analysis of the best positions on the human body for the two ECG electrodes to measure ECG signals is presented in Section 4. This is followed by the experimental results as presented in Section 5. Finally, this paper makes a conclusion in Section 6.

## 2. Wearable ECG Measurement Architecture Based on Smart Clothing

**Figure 1:**
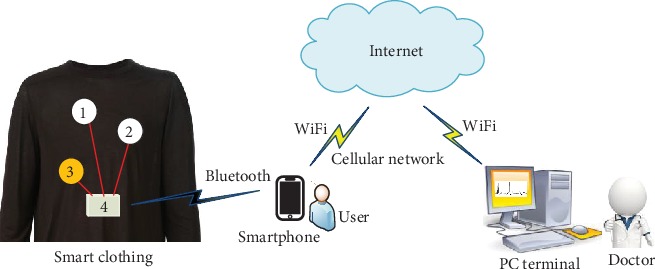
Wearable ECG measurement architecture based on smart clothing.

As a wearable measurement device, a basic requirement is that the device should be put on a human body without affecting human activities such as working, exercise, and rest. In this paper, a wearable ECG measurement architecture based on smart clothing is proposed, as described in Figure 1.

The system mainly consists of three subsystems: smart clothing, smartphone, and PC terminal. The three subsystems are connected together by some wireless communication technologies, such as WiFi, Bluetooth, cellular network, and Internet.

Some electrodes must be integrated with the smart clothing in order to measure ECG signals on the surface of the human body. These electrodes, made by conductive textile materials, should be soft and washable and should cause no irritation to the human skin, otherwise people will feel uncomfortable when wearing this kind of clothing and will not want to wear it again. Then, because the smart clothing is worn by a user in daily life to record the ECG signals for a long time, the numbers of the wearable electrodes on the smart clothing should be decreased in order that the electrodes have no effect on the users' daily life and work. And finally, the wearable electrodes should measure the ECG signals reliably.

**Figure 2:**
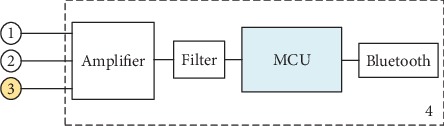
Block diagram of signal receiver.

Therefore, in our system, only three ECG textile electrodes, made by silver-coated nylon yarns, shown as three circles and marked as numbers 1, 2, and 3 in Figure 1, are knitted into the fabric of the underwear. The No. 1 and No. 2 electrodes are used to record the ECG signals on the body surface, while the No. 3 electrode fixed in the specific position on the user's body is used to obtain a reference potential that will help to reduce the common-mode interference between the two ECG signals. There is also a rectangle on the smart clothing marked as number 4 in Figure 1. It is a signal receiver that connects to the three ECG electrodes by conductive textile yarns, and it can transmit the ECG signals acquired to a smartphone. The block diagram of the signal receiver is described in Figure 2.

The signal receiver is designed based on an embedded system technology. Its components include a differential amplifier, a bandpass filter, a Microcontroller Unit (MCU), and a Bluetooth module. The differential amplifier can suppress the common-mode noise and amplify the ECG voltage from the textile electrodes. The bandpass filter has the frequency range of 0.04 Hz to 150 Hz [14] and is aimed at removing baseline drifts and power line harmonics in the ECG signals. And the MCU can reconstruct the ECG signals and then transmit the signals to a smartphone by Bluetooth. The signal receiver is powered by the minibutton battery.

The smartphone based on the IOS or Android operating systems will act as a portable device. When some special App programs designed for the processing of ECG signals are installed on the smartphone, the smartphone can display the ECG signals in real time for the user of the smart clothing, which allows the user to know his/her heart parameters anytime and anywhere. Moreover, the smartphone can immediately transmit the ECG signals received from the smart clothing to a remote PC terminal through WiFi or cellular network.

The PC terminal, however, can store large amounts of ECG signals transmitted from the smartphone for a long time. Some special programs are also designed and installed on the PC terminal in order for local or remote doctors to accurately diagnose the user's health status.

## 3. ECG Signals on Human Body

It is very important to choose where to place electrodes during measurement in order to obtain more significant ECG signals [15]. In this part of the paper, a spherical volume conductor model [16], proposed by Helmholtz, is used to describe the electric activities of the heart and to analyse the ECG signals on a human body. Although a conducting sphere is a rough approximation of the human torso, the electric potential produced by the heart in a local region can be modelled as being generated by a current dipole source inside a homogeneous conducting sphere [16, 17]. In the ECG study, interior potential tells the information of the electric source inside the body, while the potential on the surface of the sphere and the exterior potential tells the information of the electric source on the surface and the exterior of the body. The potentials enable the direct and noncontact measurement of ECG signals, and therefore, it can reflect the health status of the human heart by measurement.

**Figure 3:**
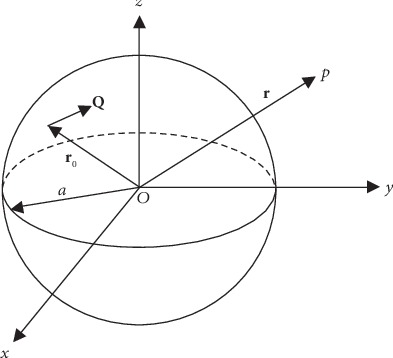
3D description of spherical volume conductor model.

For a spherical homogeneous conductor with radius *α* and conductivity *σ*, there exists a current dipole with the moment **Q** at an arbitrary location **r**_0_ inside the sphere, as shown in Figure 3.

Suppose there is a point **P** of which the radius is denoted as **r**. When point **P** is located inside the sphere, that is, |**r**| < *α*, the electric potential on point **P** can be calculated as

(1) $$\begin{array}{c} u^{-}\left(r\right)=\frac{1}{4\pi \sigma }Q∙\nabla_{r_{0}}\left[\frac{1}{\left|r-r_{0}\right|}+\frac{1}{\left|t^{2}r-r_{0}\right|}-\frac{1}{a}\ln\frac{1}{2at}\left(\hat{r}∙\left(t^{2}r-r_{0}\right)+\left|t^{2}r-r_{0}\right|\right)\right], \end{array}$$

where

(2) $$\begin{array}{c} t=\frac{a}{r}, \end{array}$$

When point **P** is located outside the sphere, that is, |**r**| > *α*, the electric potential on point **P** can be written as follows:

(3) $$\begin{array}{c} u^{+}\left(r\right)=\frac{1}{4\pi \sigma }Q\left[\frac{2\left(r-r_{0}\right)}{{\left|r-r_{0}\right|}^{3}}+\frac{\left|r-r_{0}\right|\hat{r}+\left(r-r_{0}\right)}{F\left(r,r_{0}\right)}\right], \end{array}$$

where

(4) $$\begin{array}{c} F\left(r,r_{0}\right)=r{\left|r-r_{0}\right|}^{2}+\left|r-r_{0}\right|r∙\left(r-r_{0}\right). \end{array}$$

**Figure 4:**
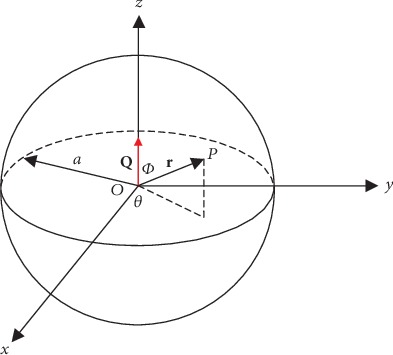
Current dipole is located in the center of the conducting sphere.

Now consider the simplest case, that is, the current dipole is located in the center of the conducting sphere, because the heart is approximately located in the middle of our chest. This case will simplify the ECG analysis with little loss of measurement accuracy. Due to the symmetric property, assume that the moment **Q** of the dipole is along the +*z* direction, as shown in Figure 4.

In the ECG study, only the electric potential on the surface of the conducting sphere is considered because in actual ECG measurements, the ECG electrodes are only attached to the skin of the human body. For any point **P** on the surface of the sphere, that is, |**r**| = *α*, the following equation is used to calculate the electric potential on point **P**, which can be seen as the ECG signals on the surface of the human body.

(5) $$\begin{array}{c} u\left(\alpha \hat{r}\right)=\frac{3}{4\pi \sigma \alpha^{2}}Q∙\hat{r}=\frac{3}{4\pi \sigma \alpha^{2}}Q\cos\emptyset , \end{array}$$

where ∅ is the angle between vector **r** and **Q**.

From equation (5), it can be seen that the electric potential on any point **P** on the surface of the human body only depends on the angle ∅ that is determined by the position of point **P**.

If two electrodes are used to measure the ECG signals on the surface of the human body and the angles of the two electrodes between vector **r** and **Q** are written as ∅_1_ and ∅_2_, respectively, the ECG signal deviation between the two electrodes can be calculated as

(6) $$\begin{array}{c} ∆u=u\left(\alpha \hat{r_{1}}\right)-u\left(\alpha \hat{r_{2}}\right)=\frac{3}{4\pi \sigma \alpha^{2}}Q\left(\cos\emptyset_{1}-\cos\emptyset_{2}\right). \end{array}$$

From equation (6), it can be seen that if the electrodes are put in the proper positions of the surface of the human body, the most significant ECG signals will be acquired.

## 4. Simulation Analysis

**Figure 5:**
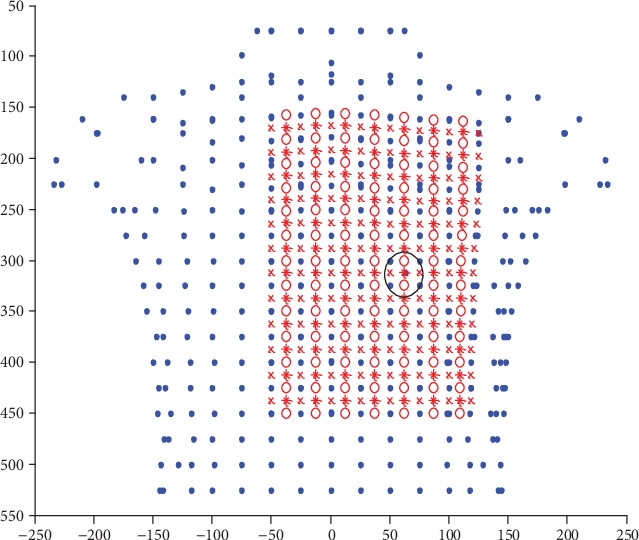
Diagram of BSPM data.

Now, in order to find the best positions for electrodes to measure ECG signals on the surface of the human body, the BSPM (body-surface potential mapping) data will be used to evaluate the value of the ECG signals. The data, consisting of ECG data for 352 torso-surface points with blue colour, were chosen from the MALT (Magnetic and Electrocardiographic Technologies) study patient population (each case with old (1-year) infarct) [18, 19], and they were provided for a single averaged PQRST complex sampled at 1 kHz, as shown in Figure 5.

Because the 352 points on the torso surface are too sparse to accurately calculate the value of ECG signals and to determine the best positions for the electrodes, another 271 points with ECG signals coloured with red around the heart were interpolated, where the points o are the average value of horizontally adjacent two points, the points x are the average value of vertically adjacent two points, and the points ∗ are the average value of adjacent four points. A round circle that covers 9 points is used to indicate an ECG textile electrode.

**Figure 6:**
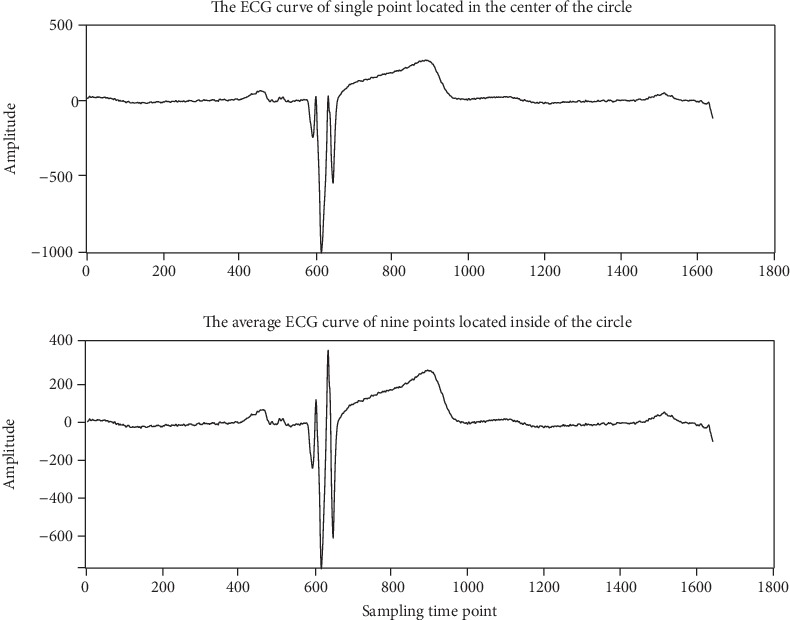
Curves of ECG values of a single point and the average value of nine points.

The curves of the ECG value of the single point located in the center of the round electrode and the ECG average value of the nine points within the round electrode are all given in Figure 6, where the *x*-axis is the sampling time points of the ECG signal and the *y*-axis is the amplitude of the ECG signal. From Figure 6, it can be seen that the curve of the ECG average value of nine points is better than that of a single point, according to the shape and peak-peak value of the ECG signals.

Now, in order to calculate the deviation of ECG signals, two round electrodes were used to measure the ECG signals. And one electrode is placed on the upper left of the human torso, and another is placed on the lower right. In order to find out the best positions for the two electrodes, the peak-peak value of ECG signals is calculated and it is used as the performance index to judge which position is better.

**Figure 7:**
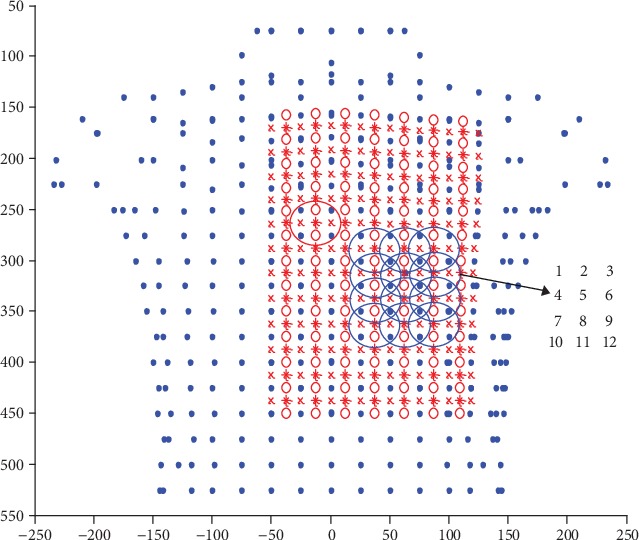
Selectable positions for the lower-right electrode.

Firstly, the position of the upper-left electrode with a red line was fixed, and 12 positions with a blue line and close to the heart were chosen as the selectable positions for the lower-right electrode. These positions were labelled as positions 1, 2,…, 12, as Figure 7 shows.

There are twelve ECG-electrode pairs between the upper-left electrode and the twelve lower-right electrodes. The deviations of the ECG values of each electrode pair were calculated, and the maximum deviation values of each ECG-electrode pair are written in Table 1, from which it can be seen that position 12 has the biggest Max deviation value among the twelve pairs. Therefore, position 12 was chosen as the best position for the lower-right electrode.

**Figure 8:**
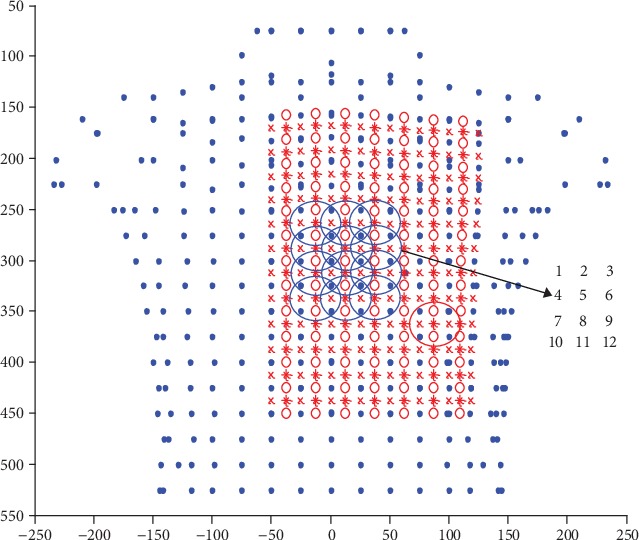
Selectable positions for the upper-left electrode.

Then, the position of the lower-right electrode was fixed in position 12 as described above, and the position of the upper-left electrode was modified. Also, 12 positions close to the heart with a dark blue line were chosen as the selectable positions for the upper-left electrode and these positions were also labelled as positions 1, 2,…, 12, as Figure 8 shows.

There are also twelve ECG-electrode pairs between the twelve upper-left electrodes and the lower-right electrode. The same as above, the deviations of the ECG values of each electrode pair were calculated, and the maximum deviation values of each ECG-electrode pair are shown in Table 2, from which it can be seen that position 11 has the biggest deviation value among the twelve pairs. Therefore, position 11 was chosen as the best position for the upper-left electrode.

Now, by the simulation of BSPM data, position 11 from the twelve upper-left positions was chosen for the upper-left electrode and position 12 from the twelve lower-right positions was chosen for the lower-right electrode, which could be the two best positions for the two textile electrodes to measure ECG signals on the surface of the human body.

## 5. Experimental Results

**Figure 9:**
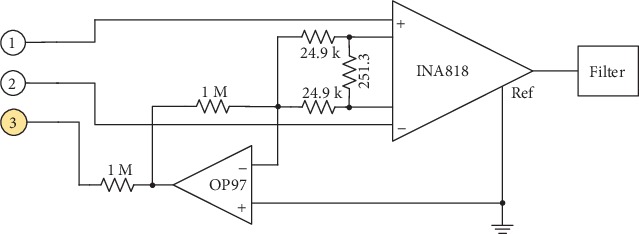
Circuit of differential amplifier.

Experiments were designed to test the performance of the proposed wearable measurement of ECG signals. The circuit of the differential amplifier of the signal receiver is designed based on chip INA818 and OP97, as Figure 9 shows. The desired gain of the amplifier is set to be 200 in our experiment.

The parts of MCU and Bluetooth of the signal receiver are designed based on IC chip nRF52840 that is an ARM Cortex-M4 32-bit processor with a Bluetooth 5, 2.4 GHz transceiver. The IC chip nRF52840 has a 12-bit 200 ksps ADC, and the size of this chip is very small, which makes it suitable for the application in the wearable devices. The ECG signals from the filter are inputted into the nRF52840 via the AIN pin of the chip. And the chip will reconstruct the ECG signals and then transmit the signals to a smartphone by Bluetooth.

**Figure 10:**
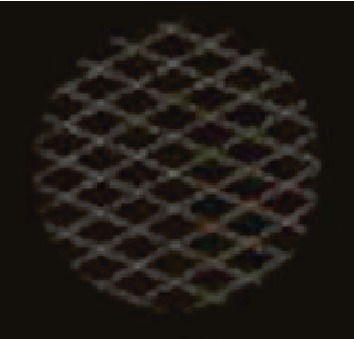
Textile electrode.

The textile electrode of the wearable measurement designed in this paper is shown in Figure 10.

**Figure 11:**
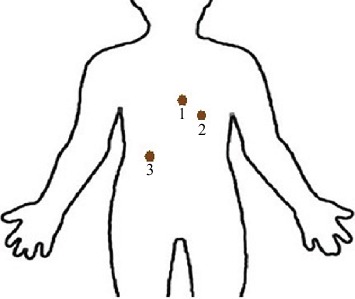
Positions of three electrodes.

There are three textile electrodes knitted into the fabric of the smart underwear, and the positions of the three textile electrodes on the human body are shown as Figure 11.

As Figure 11 shows, three conventional Ag/AgCl electrodes are also placed in the same positions to measure ECG signals, in order to compare the performance of the textile electrode to the conventional Ag/AgCl electrode. The wearable measurement using the textile electrode was tested for 5 minutes while sleeping in the supine position and 5 minutes while jogging, respectively. However, the measurement using the conventional Ag/AgCl electrode was only tested for 5 minutes while sleeping. Every measurement was repeated 5 times in the experiment in order to avoid measurement errors.

**Figure 12:**
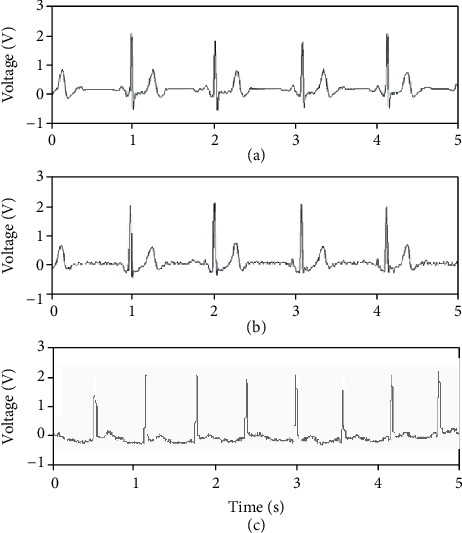
Waveforms of ECG signals.

The average values of the ECG signals from the three kinds of measurement were calculated, and the data are shown in Figure 12.

In Figure 12, Figure 12(a) is the ECG signal measured by the conventional electrode while sleeping, and Figures 12(b) and 12(c) are the ECG signals measured by the textile electrode while sleeping and jogging. Although there are some jitters in the waveform (Figure 12(c)), it is easy to recognize the *P* waves, QRS complexes, and *T* waves in the three kinds of waveforms, which means that the presented measurement can obtain good performance.

## 6. Conclusion

As people are paying more and more attention to their health, smart clothing that can measure ECG signals for a long time with high comfort and high reliability for users will meet the needs of our society, especially the older and the disabled people.

In this paper, a novel wearable ECG measurement based on smart clothing is proposed. The wearable ECG measurement system consists of three subsystems that include smart clothing, a smartphone, and a PC terminal. There are only three ECG textile electrodes knitted into the fabric of the smart clothing, and the smart clothing can transmit the acquired ECG signals to a smartphone via Bluetooth. Then, the ECG signals are sent out by the smartphone to a PC terminal via WiFi, cellular network, or Internet. In order to get more significant ECG signals, the ECG textile electrodes should be attached on the best positions of the human body. Therefore, a spherical volume conductor model is used to calculate the surface potential on the human body, and the ECG signal deviation between two electrodes is also calculated. The best positions for the electrodes to measure ECG signals is simulated by the BSPM data from the MALT group. The results show that the presented method for the measurement of ECG signals based on smart clothing can obtain good performance.

Because the ECG electrodes are made by conductive textile materials, people will feel comfortable even when they wear the smart clothing for a long time. Therefore, the proposed ECG measurement method based on smart clothing will have a promising prospect in wearable health-monitoring systems.

## Figures and Tables

**Table 1:** Max deviation of the ECG values between the upper-left electrode and every lower-right electrode.

Label	Max	Min	Max deviation
1	125.2	-222.8	348
2	597.2	-149	746.2
3	1080.1	-48.8	1128.9
4	193.2	-496.8	690
5	507.1	-281.5	788.6
6	1051.7	-135.4	1187.1
7	307.2	-698.8	1006
8	423.8	-359.5	783.3
9	1010.4	-229.7	1240.1
10	409	-837	1246
11	561.7	-391	952.7
12	1055	-269	1324

**Table 2:** Max deviation of ECG values between lower-right electrode and every upper-left electrode.

Label	Max	Min	Max deviation
1	353.6	-941.1	1294.7
2	319	-1029.8	1348.8
3	269	-1055	1324
4	243.1	-1198	1441.1
5	162.6	-1319.4	1482
6	99.2	-1447.5	1546.7
7	158.8	-1430.8	1589.6
8	75.7	-1608.7	1684.4
9	153.2	-1735.6	1888.8
10	116.5	-1557	1673.5
11	78.9	-1826	1904.9
12	158.4	-1971.7	-1813.3

## Data Availability

The data used to support the findings of this study are included within the article.

## References

[1] Majumder S., Chen L., Marinov O., Chen C.-H., Mondal T., Deen M. J. (2018). Noncontact wearable wireless ECG systems for long-term monitoring. *IEEE Reviews in Biomedical Engineering*.

[2] Disability. https://www.who.int/disabilities/en/.

[3] Ageing and health. https://www.who.int/en/news-room/fact-sheets/detail/ageing-and-health.

[4] Okada Y., Yoto T. Y., Suzuki T. A., Sakuragawa S., Mineta H., Sugiura T. Wearable ECG recorder with acceleration sensors for measuring daily stress.

[5] Park C., Chou P. H., Bai Y., Matthews R., Hibbs A. An ultra-wearable, wireless, low power ECG monitoring system.

[6] Arun C. S., Alexander A. Mobile ECG monitoring device using wearable non contact armband.

[7] Pola T., Vanhala J. Textile electrodes in ECG measurement.

[8] Vojtech L., Bortel R., Neruda M., Kozak M. (2013). Wearable textile electrodes for ECG measurement. *Advances in Electrical and Electronic Engineering*.

[9] Caldara M., Comotti D., Gaioni L. Wearable sensor system for multi-lead ECG measurement.

[10] Yoo J., Long Yan, Seulki Lee, Hyejung Kim, Hoi-Jun Yoo (2009). A wearable ECG acquisition system with compact planar-fashionable circuit board-based shirt. *IEEE Transactions on Information Technology in Biomedicine*.

[11] Lee Y.-D., Chung W.-Y. (2009). Wireless sensor network based wearable smart shirt for ubiquitous health and activity monitoring. *Sensors and Actuators B: Chemical*.

[12] Taji B., Shirmohammadi S., Groza V., Batkin I. (2014). Impact of skin-electrode interface on electrocardiogram measurements using conductive textile electrodes. *IEEE Transactions on Instrumentation and Measurement*.

[13] Paradiso R., Loriga G., Taccini N. (2005). A wearable health care system based on knitted integrated sensors. *IEEE Transactions on Information Technology in Biomedicine*.

[14] Webster J. G. (1997). *Medical Instrumentation: Application and Design*.

[15] Tao Q. A non-contact electrode for measurement of electrocardiography. http://d-scholarship.pitt.edu/10540/1/Master_Thesis_Quan_Tao_2nd_Edition.pdf.

[16] Wilson F. N., Bayley R. H. (1950). The electric field of an eccentric dipole in a homogeneous spherical conducting medium. *Circulation*.

[17] Yao D. (2000). Electric potential produced by a dipole in a homogeneous conducting sphere. *IEEE Transactions on Biomedical Engineering*.

[18] Dawoud F., Wagner G. S., Moody G., Horacek B. M. (2008). Using inverse electrocardiography to image myocardial infarction—reflecting on the 2007 PhysioNet/Computers in Cardiology Challenge. *Journal of Electrocardiology*.

[19] Goldberger A. L., Amaral L. A. N., Glass L. (2000). PhysioBank, PhysioToolkit, and PhysioNet. *Circulation*.

