# Corrigendum to “Wearable Measurement of ECG Signals Based on Smart Clothing”

**DOI:** 10.1155/2020/6437891

**Published:** 2020-11-17

**Authors:** Ming Li, Wei Xiong, Yongjian Li

**Affiliations:** ^1^Engineering Research Center of Hubei Province for Clothing Information, Wuhan Textile University, Wuhan 430200, China; ^2^Hubei Key Laboratory of Digital Textile Equipment, Wuhan Textile University, Wuhan 430200, China; ^3^Class 1112, Maple Leaf International School-Wuhan, Wuhan 410077, China

In the article titled “Wearable Measurement of ECG Signals Based on Smart Clothing” [1], the authors identified that they did not have the necessary permissions for the publication of Figures 3, 4, and 11. These figures have, therefore, been removed from the article.
